# Intra-Cycle Elastic Nonlinearity of Nitrogen-Doped Carbon Nanotube/Polymer Nanocomposites under Medium Amplitude Oscillatory Shear (MAOS) Flow

**DOI:** 10.3390/nano10071257

**Published:** 2020-06-28

**Authors:** Milad Kamkar, Soheil Sadeghi, Mohammad Arjmand, Ehsan Aliabadian, Uttandaraman Sundararaj

**Affiliations:** 1 Department of Chemical and Petroleum Engineering, University of Calgary, 2500 University Dr NW, Calgary, AB T2N 1N4, Canada; milad.kamkar1@ucalgary.ca (M.K.); ssadeghi@ucalgary.ca (S.S.); ehsan.aliabadian@ucalgary.ca (E.A.); 2 School of Engineering, University of British Columbia, Kelowna, BC V1V 1V7, Canada; mohammad.arjmand@ubc.ca

**Keywords:** nonlinear rheology, LAOS, MAOS, Nitrogen-doped CNT

## Abstract

This study seeks to unravel the effect of carbon nanotube’s physical and chemical features on the final electrical and rheological properties of polymer nanocomposites thereof. Nitrogen-doped carbon nanotubes (N-CNTs) were synthesized over two different types of catalysts, i.e., Fe and Ni, employing chemical vapor deposition. Utilizing this technique, we were able to synthesize N-CNTs with significantly different structures. As a result, remarkable differences in the network structure of the nanotubes were observed upon mixing the N-CNTs in a polyvinylidene fluoride (PVDF) matrix, which, in turn, led to drastically different electrical and rheological properties. For instance, no enhancement in the electrical conductivity of poorly-dispersed (N-CNT)_Ni_/PVDF samples was observed even at high nanotube concentrations, whereas (N-CNT)_Fe_/PVDF nanocomposites exhibited an insulative behavior at 1.0 wt%, a semi-conductive behavior at 2.0 wt%, and a conductive behavior at 2.7 wt%. In terms of rheology, the most substantial differences in the viscoelastic behavior of the systems were distinguishable in the medium amplitude oscillatory shear (MAOS) region. The stress decomposition method combined with the evaluation of the elastic and viscous third-order Chebyshev coefficients revealed a strong intra-cycle elastic nonlinearity in the MAOS region for the poorly-dispersed systems in small frequencies; however, the well-dispersed systems showed no intra-cycle nonlinearity in the MAOS region. It was shown that the MAOS elastic nonlinearity of poorly-dispersed systems stems from the confinement of N-CNT domains between the rheometer’s plates for small gap sizes comparable with the size of the agglomerates. Moreover, the intra-cycle elastic nonlinearity of poorly-dispersed systems is frequency-dependent and vanished at higher frequencies. The correlation between the microstructure and viscoelastic properties under large shear deformations provides further guidance for the fabrication of high-performance 3D-printed electrically conductive nanocomposites with precisely controllable final properties for engineering applications.

## 1. Introduction

Carbon nanotube (CNT) has emerged as one of the most popular nanofillers in the polymer composites industry since it can produce a dramatic improvement in final properties at low contents. It has been widely shown that a critical factor in controlling the electrical [1,2], mechanical [3,4,5], electromagnetic interference (EMI) shielding effectiveness [6], and rheological [7,8,9,10,11,12] properties of CNT/polymer nanocomposites (CPN) is the network structure of the nanotubes; hence, monitoring the network structure formation is of high significance. The CNT network structure can be controlled by physical (e.g., aspect ratio) or chemical (e.g., functionalization and doping) features of the individual CNTs. The doping of CNTs with other chemical elements (e.g., nitrogen) is a facile way to manipulate CNTs’ properties, which, in turn, dramatically affects the final properties of polymer nanocomposites, e.g., dielectric properties [13] and electrical conductivity [14]. For instance, nitrogen-doped CNTs (N-CNT)/polymer nanocomposites have the potential to be used for charge storage applications, such as embedded capacitors [15].

The other important factor, which controls the final properties of CNT-based polymer nanocomposites, is physical features (e.g., length and diameter) of individual CNTs. In our previous studies [15,16,17,18], employing the chemical vapor deposition (CVD) method, we were able to synthesize CNTs featuring different physical properties. The physical structures of CNTs were fine-tuned by changing the synthesis temperature or the synthesis catalyst. Significant differences in electrical conductivity, dielectric properties, EMI shielding effectiveness, and thermal properties of the CPNs were observed upon changing the physical structure of the CNTs [15,16,17,18].

As such, deep investigation of the nanocomposite microstructures holding carbon nanotubes with different features is of great importance for both academia and industry. The interrelationship between the microstructure of polymer nanocomposites and their rheological properties makes rheometry a versatile approach to study the network architecture of nanofillers. For instance, Cipiriano et al. [7] and Wu et al. [19] have studied the effects of CNT network structure on linear viscoelastic properties under small amplitude oscillatory shear (SAOS) flow by changing the aspect ratio of CNTs in polymeric matrices. However, the literature lacks fundamental studies on the nonlinear rheological behavior of nanocomposites containing nanomaterials with various structures. The nonlinear viscoelasticity of these flocculated systems can be characterized by medium and large amplitude oscillatory shear flows, known as MAOS and LAOS, respectively [20,21,22,23]. Additionally, it should be borne in mind that MAOS and LAOS tests are more applicable to the real flow fields, as in most of the polymer processing and 3D printing techniques, materials experience large and rapid deformation. 

Previously, it was shown that rheological parameters directly affect the printability of CNT-based polymer nanocomposites [24]. However, with respect to the flow behavior of 3D-printed CNT-based polymer nanocomposites, most of the works in the literature have focused on rheological properties employing simple oscillatory shear tests or flow curves. Hence, in this study, we utilized MAOS and LAOS protocols to obtain inter/intra-cycle nonlinear parameters. These parameters can provide greater insight into the characterization of complex fluids as they cover a broader domain of deformation or time scale. To unravel the underpinnings of the nonlinear responses of melted CNT-based polymeric nanocomposites under MAOS and LAOS, N-CNTs with various physical structures were synthesized. This systematic methodology provides us with a unique opportunity to investigate the effect of physical properties and dispersion characteristics of nanotubes on inter/intra-cycle nonlinear rheology of polymer nanocomposite, playing a crucial role in 3D-printing.

Using stress decomposition [25] conjugated with Chebyshev functions proposed by Ewoldt et al. [26], the nonreality of the generated polymer nanocomposites was used to assess the quality of N-CNT dispersion. In this work, we evaluated the viscoelastic behavior at extremely low angular frequencies, and we showcase the effect of jamming of the N-CNT agglomerates on Chebyshev coefficients and intra-cycle viscoelastic parameters. Microscopy imaging at micro/nanoscale coupled with AC conductivity measurement was also employed to validate the study of the network structures of N-CNTs using rheometry. 

In our previous work [8], we observed a strong intra-cycle viscous character in the MAOS region under large frequencies (e.g., 10 rad/s) as a result of the confinement of the big agglomerates of the poorly-dispersed CNTs. In this study, contrarily, we observed a strong intra-cycle elastic nonlinearity at angular frequencies as low as 0.1 rad/s. In brief, it can be asserted that this study proposes a bilateral achievement: (i) employing MAOS and LAOS techniques to obtain a better understanding of the effect of the morphology of N-CNTs on network formation within a polymer matrix and (ii) enhanced exploration of intra-cycle viscoelastic parameters in MAOS and LAOS regions using well-defined synthesized N-CNTs and nanocomposites thereof. 

## 2. Experimental

### 2.1. Materials Synthesis

The catalyst precursors used in this study were iron (III) nitrate nanohydrate and nickel (II) sulfate hexahydrate (Baker Analyzed^®^ ACS Grade). Catalysts were made by incipient wetness impregnation of catalyst precursors dissolved in distilled water on aluminum oxide support (Sasol Catalox Sba-200). The metal loading was set at 20 wt%. To prepare the catalysts for N-CNT synthesis, the catalyst particles were dried, calcinated, and reduced. N-CNTs were synthesized by carrying a combination of ethane, ammonia, and argon over the prepared catalysts in a quartz tubular reactor with a 4 cm diameter. Ethane was the carbon source, and ammonia and argon played the role of nitrogen source and inert gas carrier, respectively. The synthesis temperature and time were 750 °C and 2 h, respectively. Additional information concerning the catalyst preparation and N-CNT synthesis is detailed elsewhere [18]. 

A polyvinylidene fluoride (PVDF) matrix was procured from 3M Canada (Grade: 11008/0001), with a melting point of 160 °C. Prior to mixing, the raw materials were dried in a vacuum oven at 60 °C overnight. We used an APAM (Alberta Polymer Asymmetric Minimixer) mixer to blend synthesized N-CNTs with the PVDF matrix at 240 °C and 235 rpm. The PVDF matrix was first masticated for 3 min, and then N-CNTs were introduced into the mixing cup and mixed for an extra 14 min. The nanocomposites were generated at different N-CNT loadings, i.e., 0.5, 1.0, 2.0, and 2.7 wt%. A Carver compression molder (Carver Inc., Wabash, IN, USA) was utilized to make circular samples (25 mm diameter, 0.5 mm thickness) at 240 °C under 38 MPa pressure for 10 min for morphological, electrical, and rheological characterizations.

### 2.2. Materials Characterization

#### 2.2.1. TEM, XPS, and TGA of N-CNTs

The TEM analysis of N-CNTs was carried out on a Tecnai TF20 G2 FEG-TEM (FEI, Hillsboro, OR, USA) at a 200 kV acceleration voltage. Measurement of the geometrical features of N-CNTs was performed for 150 individual N-CNTs by means of MeasureIT software (Olympus Soft Imaging Solutions GmbH). To evaluate the impact of melt mixing on the length loss of synthesized N-CNTs, nanocomposites with 2.0 wt% N-CNT were dissolved in dimethylformamide (DMF) at 80 °C under continuous stirring, until only N-CNTs remained. Thereafter, one drop of the dispersion was mounted on a copper grid and dried in air, and the length of N-CNTs was quantified.

A Physical Electronics PHI VersaProbe 5000-XPS (ULVAC-PHI, Inc., Kanagawa, Japan) was employed to achieve XPS spectra. The spectra were acquired using a monochromatic Al source at 1486.6 eV and 49.3 W with a beam diameter of 200.0 μm. The binding energies were reported with respect to C1s at 284.8 eV. The carbon purity and crystallinity of N-CNTs were inspected employing a thermogravimetric analyzer (TGA), (TA Instruments, New Castle, DE, USA) TA instruments Q500. The N-CNT powders were heated in an air environment (Praxair AI INDK) (Praxair Inc., Danbury, CT, USA) from ambient temperature to 900 °C at a ramp rate of 10 °C/min. 

#### 2.2.2. Composite Morphology and Structure

The microdispersion state of N-CNTs within the PVDF matrix was quantified using light microscopy (LM) in transmission mode on thin cuts (5 µm thickness) of the compression-molded samples. The samples were cut with a Leica microtome RM2265 (Leica Microsystems GmbH, Wetzlar, Germany) equipped with a diamond knife. An Olympus microscope BH2 equipped with a CCD camera DP71 (both from Olympus Deutschland GmbH, Hamburg, Germany) was employed to take images from different cut sections (15 cuts, area of each: 600 × 800 µm^2^). For microdispersion state evaluation, we followed the British Standard ISO 18553:2002 method, used for the assessment of dispersion degree of pigment or carbon black in polyolefin products. The agglomerate area ratio (in %) was determined by dividing the spotted agglomerates with area equal to or larger than a circle with 5 µm diameter (area > 19.6 µm^2^) over the whole sample area. The relative transparency was enumerated by dividing the transparency of the cut over the transparency of the glass slide/cover glass assembly. 

Ultrathin sections of the samples (60 nm thickness) were cut utilizing an ultramicrotome EM UC6/FC6 (Leica, Austria) setup with an ultrasonic diamond knife at ambient temperature. Transmission electron microscopy (TEM) of the ultramicrotomed cuts was performed using TEM LIBRA 120 (Carl Zeiss SMT, Oberkochen, Germany) with an acceleration voltage of 120 kV.

*Rheology*: Rheological measurements were performed using an Anton-Paar MCR 302 rheometer (Anton Paar Gmbh, Graz, Austria) at 240 ± 0.5 °C using 25 mm cone-plate geometry with a cone angle of 1° and truncation of 47 μm and a 25 mm parallel plats geometry. Large amplitude oscillatory shear (LAOS) response of neat PVDF and N-CNT/PVDF nanocomposites was attained to characterize the origin of nonlinearities and to better understand morphologies formed by the nanofillers. In this work, we made the measurements on at least two sets of samples for each system, and we observed similar electrical and rheological behavior/values. Thus, we were able to repeat the experiments and replicated the results.

*Broadband electrical conductivity*: The broadband electrical conductivity of the nanocomposites was measured with a Bio-Logic Impedance Analyzer SP-200 EIS (BioLogic, Seyssinet-Pariset, France) in the frequency range of 10^0^ to 10^5^ Hz. The impedance analyzer was connected to a Solartron 12962 sample holder with an electrode diameter of 10 mm. The amplitude of the applied voltage was 100 mV (V_rms_~70 mV). Prior to the measurements, the electrodes were painted on the samples using silver paste.

## 3. Results and Discussion

### 3.1. Characterization of N-CNTs

The detailed characterization of N-CNTs has been provided in Supporting Information (see Figures S1–S4 and the corresponding discussion), and here we summarized the results. Based on these data, both (N-CNT)_Fe_ and (N-CNT)_Ni_ feature a bamboo-like pattern. However, they present dissimilar length, diameter, carbon purity, and quantity of nitrogen content. To be more specific, (N-CNT)_Ni_ is shorter and has lower carbon purity than (N-CNT)_Fe_. The reason behind the morphological differences of synthesized N-CNTs can be attributed to the details of the growth mechanism and is thus related to metal particle shape, carbon diffusion, or growth direction. Readers are referred to our recent work [18] for more information.

### 3.2. Characterization of Nanocomposites

#### 3.2.1. Morphology of N-CNT/PVDF Nanocomposites

We investigated the dispersion state of N-CNTs within the PVDF matrix at three different length scales. First, light microscopy (LM) was employed to illuminate the micro-dispersion state of N-CNTs. The poorly-dispersed N-CNTs at this scale are reckoned by means of the agglomerate area ratio (ISO 18553:2002 method, agglomerate area > 19.6 μm^2^). The second scale includes agglomerates with sizes equal to or slightly larger than the wavelength of visible light, ca. 400–700 nm, but smaller than visually detectable agglomerates. This type of agglomerates enhances the grey appearance of the background. Indeed, the existence of more agglomerates in this range enriches the darkness of the microtomed cut. On the smallest scale, TEM is employed to investigate the agglomerate with sizes below the LM limit. This scale reflects nano-dispersion, wherein individually-dispersed nanotubes can be recognized. 

**Figure 1:**
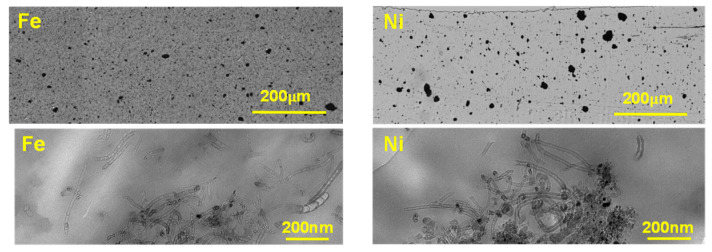
Light microscopy (LM) and TEM images of microtomed cuts of 2.0 wt% nitrogen-doped carbon nanotube/polyvinylidene fluoride (N-CNT/PVDF) nanocomposites with N-CNTs synthesized over different catalysts. LM and TEM images reveal the dispersion state at microscale and nanoscale, respectively. Figure S1 of Supporting materials shows more TEM images of the nanocomposites.

Figure 1 depicts the LM images of the microtomed cuts of the nanocomposites (5 μm thickness) with 2.0 wt% N-CNT, and Table 1 tabulates their quantification. (N-CNT)_Ni_/PVDF cut has significantly higher transparency. Quantification of the agglomerate area ratio, as presented in Table 1, explicates that the Fe-based nanocomposites show a lower agglomerate area ratio. These results vividly indicate that Fe-based N-CNTs have superior micro-dispersion compared to their Ni-based counterpart. This can be related to their superior physical features.

The TEM examination clears discrepancies between the nanocomposites by inspecting nano-dispersion state of N-CNTs (Figure 1). The nanocomposite holding (N-CNT)_Ni_ presents inferior nano-dispersion, where agglomerated areas and polymer-rich areas are quite segregated and evident. In addition, the dark black spots, which are catalyst particles, are noticeable, denoting the low carbon purity of (N-CNT)_Ni_. TEM images are in accord with LM images, both of which imply inferior dispersion of (N-CNT)_Ni_/PVDF nanocomposites compared to (N-CNT)_Fe_/PVDF nanocomposites.

#### 3.2.2. Broadband Electrical Conductivity of N-CNT/PVDF Nanocomposites

The broadband electrical conductivity is comprised of a frequency-dependent and a frequency-independent part. The frequency-independent part derives from DC conductivity, movement of free charges in phase with the applied electric field. The frequency-dependent part originates from the reorientation of electric dipoles in each half cycle of the alternating field. Broadband electrical conductivity of insulative materials has an ascending trend with frequency (AC conductivity prevails), whereas that of conductive materials is frequency independent (DC conductivity dominates). In semi-conductive materials, there is a critical frequency below which DC current prevails, whereas, above that frequency, AC current is dominant [13,15,27]. Thus, broadband electrical conductivity can be employed as a sensitive parameter to examine the level of conductive network formation in conductive polymer nanocomposites.

**Figure 2:**
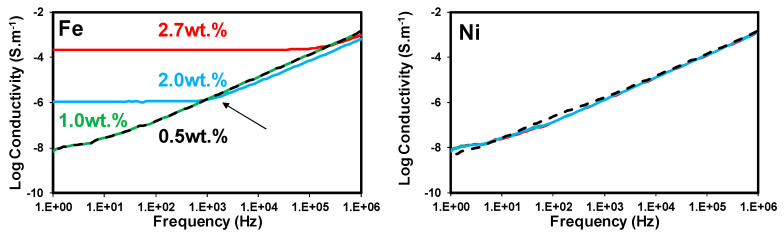
Broadband electrical conductivity of N-CNT/PVDF nanocomposites with different N-CNT loadings. N-CNTs were synthesized over Fe and Ni catalysts. Reprinted with permission from Ref. [15], with permission from Elsevier, 2020.

Figure 2 depicts the broadband electrical conductivity of the generated nanocomposites over a wide frequency range (10^0^–10^5^ Hz). The developed nanocomposites presented very different broadband electrical conductivities, implying the significant impact of the growth catalyst on the level of conductive network formation in N-CNT/PVDF nanocomposites. The nanocomposites (N-CNT)_Fe_ indicated an insulative behavior at 1.0 wt%, a semi-conductive behavior at 2.0 wt%, and a conductive behavior at 2.7 wt%. It was also observed that the Ni-based nanocomposites presented insulative behavior over the whole investigated concentration range, suggesting that no conductive network was formed in the Ni-based nanocomposites.

The electrical conductivity of carbon nanotube/polymer nanocomposites relies on many factors such as the content, intrinsic conductivity, carbon purity, and aspect ratio of carbon nanotubes, the inherent properties of the polymer matrix, the quality of the interaction between the carbon nanotube and polymer matrix, the dispersion state of the carbon nanotube, the mixing technique, the crystallinity of the matrix, etc. [28,29]. It has been reported that well-dispersed CNTs with a higher aspect ratio (larger length and smaller diameter) present a lower percolation threshold and enhanced electrical properties [30,31]. Thus, the superior electrical performance of the (N-CNT)_Fe_/PVDF nanocomposites can be related to the high aspect ratio, high carbon purity, and good dispersion state of (N-CNT)_Fe_ compared to (N-CNTs)_Ni_ (see Figure 1). In the case of (N-CNT)_Ni_, it can be asserted that its smaller diameter and thus larger surface area were counterbalanced with its smaller length, poor carbon purity, and significantly worse state of dispersion (see Figure 1).

#### 3.2.3. Viscoelastic Properties of N-CNT/PVDF Nanocomposites 

The short-range structural factors can merely affect the final macroscopic performance of the CNT-based polymer nanocomposites. The macroscopic properties of polymer nanocomposites mainly depend on the mesoscopic network structure of the nanofillers, which is constructed by long-range interaction through the polymeric matrix. It has been widely shown that the viscoelastic behavior of the materials is sensitive to the long-range interaction between nanofillers [19,32]. To study the long-range structural factor, i.e., the nanofiller superstructure, rheological response of the nanocomposite samples under different flow conditions in both linear and nonlinear regions are intensely studied in this section. 

**Figure 3:**
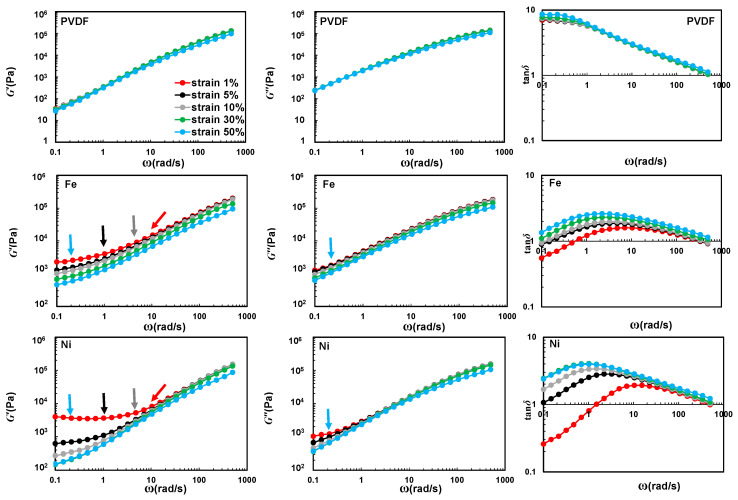
Dynamic moduli and damping factor of neat PVDF and N-CNT/PVDF nanocomposites containing 2.0 wt% of carbon nanotubes synthesized on different catalysts for strain amplitudes of γ_0_ = 1, 5, 10, 30, and 50% using cone-plate geometry (truncation of 47 μm) at 240 °C. Data points highlighted by arrows will be compared with first order elastic $e_{1}$ and viscous $v_{1}$ Chebyshev coefficients, respectively.

Figure 3 shows the dynamic storage modulus (*G*′), loss modulus (*G*″), and damping factor (tanδ) as a function of frequency in the range of SAOS to MAOS regions (γ_0_ = 1, 5, 10, 30, and 50%). In this test, the frequency was swept from 0.1 to 625 rad/s at 240 °C. According to Figure 3, it is clear that the addition of synthesized N-CNTs remarkably improves the low-frequency dynamic storage modulus (*G*′) and decreases the frequency dependency of the dynamic moduli. This nonterminal behavior represents that large-scale (i.e., reptation) relaxation of the polymer phase is restrained strongly by the presence of N-CNTs in a well-dispersed (N-CNT)_Fe_/PVDF system. 

It is well-known that a better dispersion quality leads to greater improvement in rheological properties. For instance, in our previous works [9,11], we observed a higher storage modulus for PVDF/CNT and PS/CNT systems by improving the dispersion quality of nanotubes. Despite the previous observations, in this work, we can recognize a higher *G*′ value and lower tanδ for the poorly-dispersed (N-CNT)_Ni_/PVDF compared to the well-dispersed (N-CNT)_Fe_/PVDF system in the SAOS region (γ_0_ = 1%). The higher *G*′ compared to *G*″, leading to a tanδ < 1 condition for poorly-dispersed (N-CNT)_Ni_/PVDF highlights an intensified elastic behavior. 

The elastic behavior (i.e., *G*′ > *G*″) of well-dispersed (N-CNT)_Fe_/PVDF nanocomposites can be correlated to the formation of a 3D percolated nanofiller network at the corresponding nanotube content (2.0 wt.%). For poorly-dispersed (N-CNT)_Ni_/PVDF nanocomposites, this behavior is a direct consequence of confinement of a large number of gap-spanning aggregates with sizes comparable to the gap size of the cone plate geometry used in this study (the truncation at the cone tip is 47.0 μm and the gap size increases to roughly 265 μm at the edge of the loaded sample). This hypothesis is in line with microstructural parameters obtained from optical microscopy and electrical conductivity data. As can be seen in Figure 2, (N-CNT)_Ni_-based nanocomposites presented insulative behavior over the entire investigated range of CNT loading, suggesting that no interconnected conductive network was formed in the (N-CNT)_Ni_-based nanocomposites; this is in line with optical (large agglomerates and high transparency) and TEM images (low number of individual CNTs). However, the (N-CNT)_Fe_/PVDF samples showed an insulative behavior at 1.0 wt%, a semi-conductive behavior at 2.0 wt%, and a conductive behavior at 2.7 wt%, indicative of the formation of an interconnected network structure at (N-CNT)_Fe_ concentrations equal to or greater than 2.0 wt%.

In our recent work [8], we observed different resilience of CNT structures to yielding with increasing the strain amplitude in the frequency sweep test for well- and poorly-dispersed systems. Hence, to obtain further insight into the microstructure of the N-CNT/PVDF nanocomposites, the frequency sweep test was conducted at higher strain amplitudes (γ_0_ = 5, 10, 30, and 50%, see Figure 3). By increasing the amplitude of deformation, both *G*′ and *G*″ of all samples decreased (except for neat PVDF). Upon increasing the amplitude of deformation, the rate of the drop in *G*′ is faster than *G*″, resulting in a ramping trend in damping factor (tanδ). This behavior is due to the rupture of the nanofillers’ structures. However, samples containing different types of N-CNT behave differently in response to the increase in the value of the input strain amplitude. That is, a dramatic decrease in the value of the *G*′ (about an order of magnitude) accompanied by a sudden increase in the value of the damping factor (tanδ) to values greater than 1.0 at low frequencies for (N-CNT)_Ni_/PVDF nanocomposites were observed by a small increase in the input strain amplitude (from γ_0_ = 1% to 5%). This reveals a solid-to-liquid-like behavior (*G*″ > *G*′) transition for this sample. However, a moderate decrease in the *G*′ value of the (N-CNT)_Fe_/PVDF nanocomposites is observable, and the damping factor (tanδ) is still smaller than 1.0 at low frequencies at the strain amplitude of γ_0_ = 5%. These findings reveal different resilience of the nanofiller network structure against the applied shear flow for each type of N-CNT, which potentially stems from the differences in the dispersion quality of the (N-CNT)_Fe_ and (N-CNT)_Ni_ in the PVDF matrix. 

As mentioned earlier, the poorly-dispersed (N-CNT)_Ni_ throughout the PVDF matrix forms large-sized isolated agglomerates with deprived interconnectivity. Hence, a weak nanofiller network structure of the poorly-dispersed (N-CNT)_Ni_ forms under the confinement of rheometer plates, which demolishes under a weak oscillatory deformation field (γ_0_ = 5%). In contrast, (N-CNT)_Fe_ forms a much stronger 3D interconnected network as a result of a better micro- and nano-dispersion state. Hence, its network structure survives up to higher strain amplitudes. To get further insight into the network structure of the nanocomposites, the viscoelastic response of the samples is investigated more deeply under MAOS and LAOS flow in the next section.

**Figure 4:**
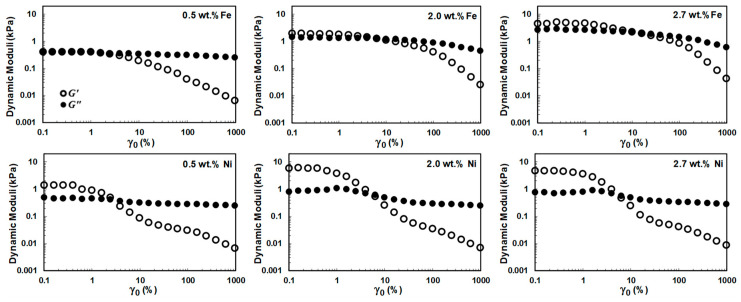
Oscillatory amplitude sweep response of neat PVDF and CNT/PVDF nanocomposites containing 0.5, 2.0, and 2.7 wt.% of carbon nanotubes synthesized on different catalysts for strain amplitudes of γ_0_ = 0.1–1000% at an angular frequency of ω = 0.1 rad/s using cone-plate geometry (truncation of 47 μm) at 240 °C.

Figure 4 shows the oscillatory amplitude sweep response of the nanocomposites over a wide range of applied strain amplitudes from γ_0_ = 0.001 (0.1%) to 10 (1000%) at a fixed angular frequency of ω = 0.1 rad/s. The initial plateau region of the dynamic strain sweep experiment, i.e., where the elastic modulus is independent of input strain amplitude, is identified as linear viscoelastic region (LVR). As the strain amplitude exceeds the limit of the LVR, above the critical strain amplitude (strain amplitude at which linear-to-nonlinear transition occurs), *G*′ decreases for all samples. The drastic drop in *G*′ and the appearance of the nonlinearity in the viscoelastic response of well-dispersed polymer nanocomposites is due to partial rupture of the hybrid network formed by polymer-polymer, polymer–filler (interfacial interactions), and filler-filler interactions (nanotubes interlocking). This results in a decrease in interconnectivity within the system, leading to a decline in elastic character and emergence of inter-cycle strain softening. 

As can be seen in Figure 4, the value of the low-strain plateau moduli, the limit of the LVR, and the critical strain amplitude are different for each (N-CNT)-type polymer nanocomposite, and more importantly, they fall into the nonlinear viscoelastic region with remarkably different patterns. These differences in viscoelastic behavior of well-dispersed and poorly-dispersed systems under SAOS and MAOS flows show that both linear and nonlinear viscoelastic responses of (N-CNT)-based nanocomposites are rigorously sensitive to the nanofillers’ physical structure and, thus, the resulting microstructure.

Based on Figure 4, the yielding process is multi-step for all concentrations of poorly-dispersed (N-CNT)_Ni_/PVDF nanocomposites, and there is no enhancement in the value of the *G*′ by increasing the (N-CNT)_Ni_ nanofiller content from 2.0 to 2.7 wt.% (see Figure S5 for better resolution). However, (N-CNT)_Fe_/PVDF nanocomposite follows a single-step yielding at all concentrations. Additionally, contrary to the (N-CNT)_Ni_/PVDF nanocomposites, the storage modulus remarkably enhances for (N-CNT)_Fe_-based nanocomposites by increasing the filler concentration from 2.0 to 2.7 wt.% (see Figure S5). It has been shown that the elastic modulus of the filled systems increased because of the immobilized polymer chains entrapped inside the nanofiller network. These entrapped chains behave as part of the un-deformable rigid fillers, increasing the effective filler volume fraction, termed the ‘hydrodynamic effect’ [33]. Hence, increasing the N-CNT content in well-dispersed systems ((N-CNT)_Fe_/PVDF) leads to an increase in the volume fraction of entrapped polymer, and this enhances *G*′.

The multi-step yielding process has been observed for a wide range of systems (e.g., nanoemulsions [34], glassy microgels [35], and polymer nanocomposites [36]). Several mechanisms, such as drainage and expulsion of interstitial fluid within the fractal clusters as a result of compression of particle-rich domains, have been proposed for the first stage of yielding in multi-step yielding transition. In our previous work [36], we compared the network structure of polymer nanocomposites containing two nanofillers featuring different geometries (CNT and graphene nanoribbon (GNR)). A single-step yielding process was observed for well-dispersed percolated CNT, while the yielding process for GNR/polymer nanocomposites at low and intermediate concentrations was multi-step, accompanied by a maximum of *G*″. The pre-yielding process in GNR/polymer nanocomposite was attributed to the initial flow-induced structural reorganization, pairwise rotation, and densification of network clusters. 

In another work [8] we showcased that the first step of yielding in the MAOS region for poorly-dispersed CNT-based polymer nanocomposites can be attributed to the breakage of the nanofiller network structure formed under the confinement of rheometer plates. As a result of further increase in the input strain amplitude, this initial yielding pattern in the MAOS region was followed by a secondary yielding process in the LAOS region. It has been shown that the second yielding stems from the nature of the polymeric medium, which is related to the widespread yielding of the polymer matrix in the LAOS region. Hence, due to the above-mentioned reasons, the poorly-dispersed (N-CNT)_Ni_/PVDF systems, containing confined large-sized agglomerates of nanofillers, go through a pre-yielding process at intermediate strain amplitudes followed by widespread bond rupture and breakage of the compressed particle-rich agglomerates conjugated with yielding of the polymer matrix under large strain amplitudes (second yielding). 

To further verify this hypothesis, the strain amplitude was swept at larger gap sizes (see Figure S6). The following characteristics were observed upon increasing the gap size: (1) the low-strain plateau moduli of poorly-dispersed (N-CNT)_Ni_/PVDF systems significantly decreased to lower values (compare Figure 4 and Figure S6), (2) the pre-yielding pattern in the MAOS region vanishes, (3) there is no enhancement in the dynamic moduli by increasing the (N-CNT)_Ni_ content from 0.5 to 2.0 wt%, and (4) *G*′ <G″ in the probed strain amplitude window. These findings clearly indicate that (N-CNTs)_Ni_ could not effectively form a network structure, even at a nanotube concentration as high as 2.0 wt.%. Thus, the intensified solid-like behavior of poorly-dispersed (N-CNT)_Ni_/PVDF systems observed in Figure 4 is because of the bridges of nanofillers aggregates spanning the rheometer’s gap. 

However, the single-step yielding of nanocomposites containing (N-CNT)_Fe_ implies a “stochastic erosion” [37] mechanism. The following sections will provide more in-depth information on the origin of the first yielding and intra-cycle nonlinearity in N-CNT/PVDF nanocomposites based on the qualitative Lissajous-Bowditch plots and quantitative nonlinear Chebyshev parameters obtained in the MAOS region. 

#### 3.2.4. Lissajous-Bowditch Plots

To investigate the differences in the nonlinear behavior of different N-CNT-type nanocomposites observed in Figure 4, we used the stress decomposition method, which decomposes stress into elastic (σ′) and viscous (σ″) contributions. Using this method, we were able to analyze the distorted output stress waveform in the nonlinear region. Based on this concept, the elastic stress contribution can be expressed by an odd function of strain or even function of strain rate, whereas the viscous stress contribution should be even in strain and odd in strain rate [25,26]. The visual depiction of total shear stress ($\sigma =\sigma^{\prime }+\sigma \prime \prime$) as a function of strain or the strain rate in one cycle can be presented using Lissajous-Bowditch loops (will be called Lissajous loops for simplification). Figure S7 shows the schematics of the Lissajous loops for perfectly elastic and pure viscous materials. Since the input strain deformation and output shear stress waveforms superimpose for a perfectly elastic system, the Lissajous loops would be a line in elastic projection and a circle in viscous projection (see Figure S7a), whereas this behavior is exactly opposite for a pure viscous system, as the output shear stress is in phase with shear rate (see Figure S7b). Thus, based on the viscoelastic concept, an ellipsoidal shaped Lissajous loop is expected in both projections for viscoelastic materials in the linear viscoelastic framework.

**Figure 5:**
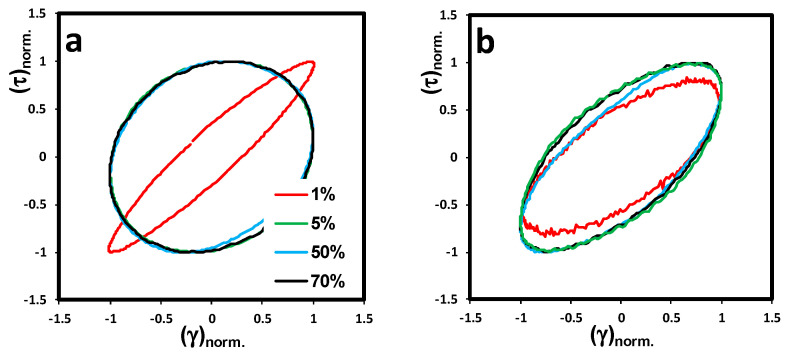
Normalized elastic Lissajous-Bowditch loops of N-CNT/PVDF nanocomposites containing 2.0 wt% of carbon nanotubes synthesized on (**a**) Ni and (**b**) Fe catalysts using cone-plate geometry (truncation of 47 μm) at 240 °C. Projections on the elastic (τ-γ) planes are presented at strain amplitudes of $\gamma_{0}$ = 1.0, 5.0, 50, and 70% and an angular frequency of ω = 0.1 rad/s.

**Figure 6:**
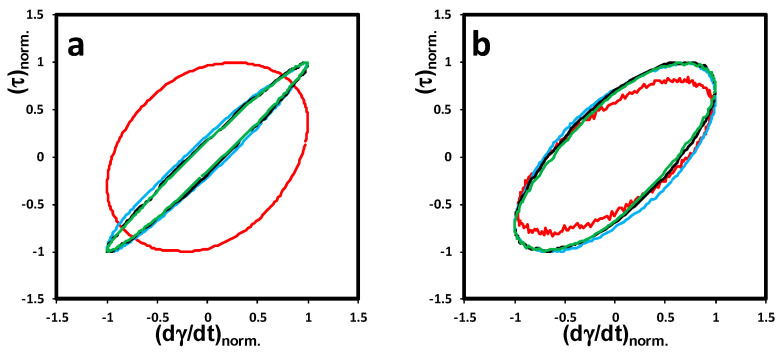
Normalized viscous Lissajous-Bowditch loops of N-CNT/PVDF nanocomposites containing 2.0 wt% of carbon nanotubes synthesized on (**a**) Ni and (**b**) Fe catalysts using cone-plate geometry (truncation of 47 μm) at 240 °C. Projections on the viscous (τ-$\dot{\gamma }$) planes are presented at strain amplitudes of $\gamma_{0}$ = 1.0, 5.0, 50, and 70% and an angular frequency of ω= 0.1 rad/s.

The normalized elastic and viscous Lissajous loops of N-CNT nanocomposites are plotted in Figure 5 and Figure 6, respectively, at strain amplitudes of γ_0_ = 1, 5, 50, and 70% and an angular frequency of ω = 0.1 rad/s. The slope of the major axis and the area of the ellipses are different for each N-CNT-type nanocomposite. This verifies the sensitivity of the Lissajous loops to any changes in microstructure. As can be seen in Figure 5a, the elastic Lissajous loop of (N-CNT)_Ni_/PVDF at a strain amplitude of 1% tends to be narrower with a higher slope of the major axis of the ellipse compared to (N-CNT)_Fe_/PVDF. This behavior indicates a more solid-like response in the viscoelastic character of (N-CNT)_Ni_/PVDF, which can be correlated to the bridges of nanofillers spanning the rheometer’s gap. This elastic-dominant response of (N-CNT)_Ni_/PVDF disappears by increasing the gap size of the rheometer’s plates and running the test using parallel-plate geometry at a gap size of 200 μm (compare Figure S8a and Figure 5a). Based on Figure S8a, the elastic Lissajous loop at γ_0_ = 1% becomes considerably broader, and the slope of the major axis of the ellipse dramatically decreases, revealing a liquid-like response in larger gap sizes for (N-CNT)_Ni_/PVDF. 

As the strain amplitude increases (γ_0_ > 5%), all the Lissajous loops superimpose (see Figure 5a). This supports the strain amplitude results in Figure 4, where the first step of yielding was observed in the MAOS region. Hence, in the MAOS region, the gap-spanning structures in (N-CNT)_Ni_/PVDF are broken down by the applied deformation, and the Lissajous loops converge to the limiting case of the PVDF matrix. In contrast, as shown in Figure 5 and Figure 6, (N-CNT)_Fe_/PVDF is more resilient to the applied deformation. That is, (N-CNT)_Fe_/PVDF maintains its elastic character, originating from the 3D network structure of the nanotubes at larger deformations. Thus, as opposed to the SAOS region, elastic Lissajous plots are narrower for (N-CNT)_Fe_/PVDF compared to (N-CNT)_Ni_/PVDF in the MAOS region (compare Figure 5a,b).

As the input strain amplitude increases, the occurrence of nonlinearity in a viscoelastic system can be discerned by any distortion in the ellipsoidal shape of Lissajous loops. At strain amplitudes higher than 1%, (N-CNT)_Ni_/PVDF polymer nanocomposites fall into the nonlinear viscoelastic region (see the critical strain amplitudes in Figure 4, the onset of the decrease in storage modulus (*G*′)). Therefore, because of the excitation of the higher harmonics in the output shear stress waveform, we expect the appearance of the distortions in Lissajous loops in the MAOS region. However, no evident distortion can be discerned by the naked eye in the Lissajous loops of our samples in the MAOS region. This makes the interpretation of the nonlinearity based on Lissajous loops challenging. Hence, we further investigate the elastic nonlinearity of the prepared nanocomposites using qualitative intra-cycle nonlinear parameters in the next sections. 

#### 3.2.5. Elastic Intra-Cycle Nonlinearity Index (*S*) and Nonlinear Viscoelastic Measures of $G_{L}^{\prime }$ and $G_{M}^{\prime }$

Comparing the local viscoelastic moduli (i.e., large-strain modulus (${\frac{\sigma }{\gamma }|}_{\gamma =\pm \gamma_{0}}\equiv G_{L}^{\prime }$) and minimum-strain modulus (${\frac{d\sigma }{d\gamma }|}_{\gamma =0}\equiv G_{M}^{\prime }$)) can assist in interpreting the intra-cycle elastic nonlinear behavior of the materials [26]. It is noted that both $G_{M}^{\prime }$ and $G_{L}^{\prime }$ converge to the linear elastic modulus in the linear viscoelastic region, i.e., $G_{M}^{\prime }$ = $G_{L}^{\prime }$ = $G_{1}^{\prime }$ = $G^{\prime }$. Detailed explanation of these parameters can be found in our recent work [20]. These elastic measures have been used by Ewoldt et al. [26,38] to develop a dimensionless index for interpretation of intra-cycle elastic nonlinearity defined as:

(1) $$S\equiv \frac{G_{L}^{\prime }-G_{M}^{\prime }}{G_{L}^{\prime }}.$$

where an *S* (strain stiffening ratio) value equal to 0 corresponds to a linear viscoelastic response, a positive *S* indicates intra-cycle strain-stiffening behavior, and a negative *S* is indicative of intra-cycle strain-softening.

**Figure 7:**
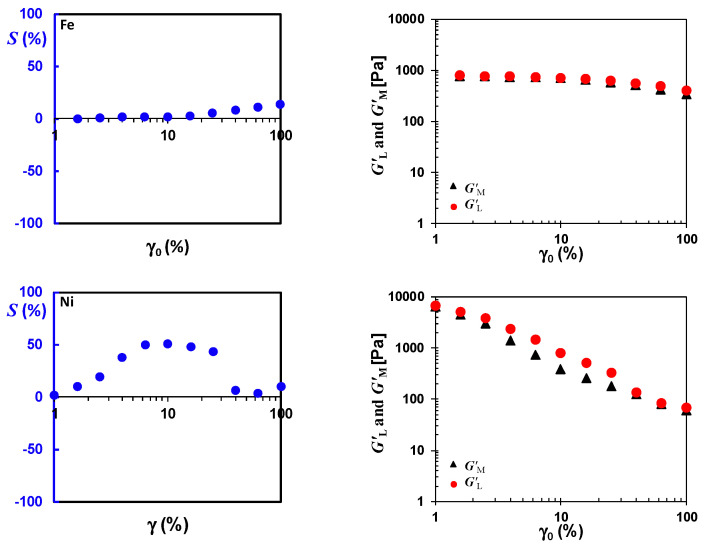
Elastic (*S*) intra-cycle nonlinearity index and nonlinear viscoelastic measures of $G_{L}^{\prime }$ and $G_{M}^{\prime }$ as a function of imposed strain amplitude for (N-CNT)_Fe_/PVDF and (N-CNT)_Ni_/PVDF nanocomposites containing 2.0 wt% of carbon nanotubes using cone-plate geometry (a truncation of 47 μm) at an angular frequency of ω = 0.1 rad/s at 240 °C.

Figure 7 shows the intra-cycle nonlinear parameters obtained under multiple deformations in the MAOS region for (N-CNT)_Fe_/PVDF and (N-CNT)_Ni_/PVDF nanocomposites containing 2.0 wt% of nanotubes. $G_{M}^{\prime }$ and $G_{L}^{\prime }$ are roughly constant for (N-CNT)_Fe_/PVDF nanocomposites, and the *S* index is close to zero for a strain amplitude up to 40%. These results can be interpreted as no elastic inter- and intra-cycle nonlinearity occurs in well-dispersed samples up to a strain amplitude of 40%. This is in line with Figure S12, in which third-order elastic Chebyshev coefficients are extremely small for (N-CNT)_Fe_/PVDF. However, it is apparent that $G_{M}^{\prime }$ and $G_{L}^{\prime }$ of the (N-CNT)_Ni_/PVDF nanocomposites are not constant even in the SAOS/MAOS regions (γ < 10%). The simultaneous decreasing trend of the $G_{M}^{\prime }$ and $G_{L}^{\prime }$ of the (N-CNT)_Ni_/PVDF nanocomposite can be interpreted as inter-cycle strain-softening behavior in the MAOS regions. Moreover, the rate of the decrease of the $G_{M}^{\prime }$ is greater than the rate of the drop in $G_{L}^{\prime }$, resulting in a positive *S* index (intra-cycle strain stiffening behavior). This behavior supports the results of Figure S12. That is, in line with Figure 7, the third-order elastic Chebyshev coefficient of (N-CNT)_Ni_/PVDF nanocomposite increases up to a maximum value at a strain amplitude of γ_0_ = 5% and then decreases as a result of a further increase in strain amplitude (see Figure S12). 

As can be seen in Figure S9, the nonlinear inter and intra-cycle nonlinear behavior of poorly-dispersed (N-CNT)_Ni_/PVDF nanocomposite disappeared by increasing the gap size of the plates of the rheometer to 200 μm (compare Figure S9 and Figure 7). This further confirms that the observed elastic nonlinearity in the poorly-dispersed (N-CNT)_Ni_-based polymer nanocomposites is because of the confinement of the CNT’s agglomerates between the rheometer plates in small gaps. Interestingly, the observed intra-cycle strain-stiffening behavior in the MAOS region is frequency-dependent and vanishes by increasing the frequency of deformation to ω = 10 rad/s (see Figure S10). However, the inter-cycle nonlinearity at ω = 10 rad/s still features two-step yielding (strain-softening in the MAOS region, see *G′* in Figure S10). This is in complete agreement with our recent work [8] in which no elastic intra-cycle nonlinearity was observed in the MAOS region for poorly-dispersed systems featuring a two-step yielding process at ω = 10 rad/s.

It should be mentioned that the observed intra-cycle elastic nonlinearity in this work in the MOAS region does not contradict our previous observations [8], in which the poorly-dispersed system did not show any intra-cycle elastic nonlinearity [8]. This can be better understood if one considers the *S* index in Figure S10 of Supplementary Materials. As can be seen in Figure S10, there is no intra-cycle elastic nonlinearity (i.e., the *S* index is roughly zero) in the poorly-dispersed (N-CNT)_Ni_/PVDF nanocomposite at an angular frequency of ω = 10 rad/s. 

Hence, the results of this paper and our previous work [8] show (1) the severe sensitivity of the intra-cycle viscoelastic nonlinear parameters to any changes in microstructure, (2) the intra-cycle elastic nonlinearity of the poorly-dispersed systems caused by the confinement of the rheometer’s plates in the MAOS region is frequency-dependent, and it vanishes upon increasing the frequency, and (3) the higher sensitivity of intra-cycle nonlinear behavior to the frequency of deformation compared to inter-cycle nonlinear behavior.

## 4. Conclusions

The current study showed that the synthesis catalyst (e.g., Fe and Ni) has a substantial impact on the physical and chemical properties of nitrogen-doped carbon nanotubes (N-CNTs). Employing TEM, XPS, and TGA techniques, we obtained information about the diameter, length, crystallinity, carbon purity, nitrogen content, and nitrogen-bonding type of synthesized N-CNTs. The morphological and electrical study of the generated nanocomposites showed better dispersion quality for (N-CNT)_Fe_/polyvinylidene fluoride (PVDF) as compared to (N-CNT)_Ni_/PVDF. 

To get further insight into the microstructure of the N-CNT-based polymer nanocomposites, their viscoelastic behavior was studied using linear and nonlinear rheology. Employing a frequency sweep at multiple deformations revealed the drastically distinct resilience of each type of N-CNT structure to yielding with increasing the strain amplitude. Afterward, a strain amplitude sweep test was conducted on the samples. Comparing the lumped viscoelastic parameters (e.g., storage modulus G′) of (N-CNT)_Fe_/PVDF and (N-CNT)_Ni_/PVDF verified that the most pronounced difference in the viscoelastic response of the samples occurs in the medium amplitude oscillatory shear (MOAS) region. Hence, we explored the origin of dissimilar nonlinearities in the MOAS region further with the aid of qualitative and quantitative intra-cycle nonlinear measures (e.g., Chebyshev coefficients) by adopting stress decomposition. A strong intra-cycle strain stiffening and inter-cycle strain-softening behavior was observed for the poorly-dispersed (N-CNT)_Ni_/PVDF sample in the MAOS framework, whereas no nonlinearity was observed for well-dispersed (N-CNT)_Fe_/PVDF nanocomposites. It was also shown that the nonlinearity of poorly dispersed systems is frequency and gap size dependent. 

The rheological methods used in this work can distinguish different viscoelastic behavior in the nonlinear region, which, in turn, provides a robust framework to inspect microstructural differences in polymer nanocomposites. Moreover, since nonlinear rheology can simulate real flow conditions in polymer processing techniques and 3D printing, the physical insights provided by nonlinear rheological investigation of polymeric nanocomposite samples can be used to optimize processing conditions in a real flow.

## Figures and Tables

**Table 1:** Agglomerate area ratio and relative transparency of thin cuts of 2.0 wt% N-CNT/PVDF nanocomposites with N-CNTs synthesized over different catalysts.

Catalyst	Fe	Ni
Agglomerate Area Ratio (%)	1.8	2.8
Relative Transparency (%)	53	86

## Supplementary Materials

The following are available online at https://www.mdpi.com/2079-4991/10/7/1257/s1, Figure S1: High-resolution TEM micrographs of N-CNTs grown over Fe and Ni catalysts. Figure S2: Average diameter and length of N-CNTs synthesized over various catalysts. Figure S3: Main types of nitrogen bonding in N-CNTs. Figure S4: TGA and DTG plots of N-CNTs grown over different catalysts. Figure S5: Los-strain plateau modulus for poorly and well-dispersed CNT/PVDF nanocomposites. Figure S6: Oscillatory amplitude sweep response of (N-CNT)_Ni_/PVDF nanocomposites. Figure S7: Schematic of Lissajous-Bowditch loops for (a) a perfect elastic and (b) a perfect viscous material on different projections. Figure S8: (a) Elastic and (b) viscous Lissajous-Bowditch loops (N-CNT)_Ni_/PVDF nanocomposites containing 2.0wt% of carbon nanotubes using a parallel-plate geometry. Figure S9: Elastic (*S*) intra-cycle nonlinearity index and nonlinear viscoelastic measures of $G_{L}^{\prime }$ and $G_{M}^{\prime }$ as a function of imposed strain amplitude for (N-CNT)_Ni_/PVDF nanocomposites containing 2.0wt% of carbon nanotubes using a parallel-plate geometry. Figure S10: Elastic (*S*) intra-cycle nonlinearity index, nonlinear viscoelastic measures of $G_{L}^{\prime }$ and $G_{M}^{\prime }$ and *G′* as a function of imposed strain amplitude for and (N-CNT)_Ni_/PVDF nanocomposites using cone-plate geometry (truncation of 47 μm) at an angular frequency of ω=10 rad/s at 240 °C. Figure S11: First order elastic Chebyshev coefficient *e*_1_ for N-CNT/PVDF nanocomposites. Figure S12: Intra-cycle elastic nonlinearity measures: normalized third-order Chebyshev coefficient *e*_3_/*e*_1_ for CNT/PVDF nanocomposites. Table S1: Nitrogen content and percentages of various types of nitrogen bonding for N-CNTs synthesized over Fe and Ni catalysts.
