# Supplementary material for: Intra-Cycle Elastic Nonlinearity of Nitrogen-Doped Carbon Nanotube/Polymer Nanocomposites under Medium Amplitude Oscillatory Shear (MAOS) Flow
Source: Nanomaterials (Basel). 2020 Jun 28;10(7):1257. doi: 10.3390/nano10071257 (PMC7407161; doi:10.3390/nano10071257)
Supplement: Supplementary file 1 [file nanomaterials-10-01257-s001.pdf]

Supporting Information

Intra-Cycle Elastic Nonlinearity of Nitrogen-Doped Carbon Nanotube/Polymer Nanocomposites under Medium Amplitude Oscillatory Shear (MAOS) Flow

Milad Kamkar [1], Soheil Sadeghi [1,†], Mohammad Arjmand [2], Ehsan Aliabadian [1] and Uttandaraman Sundararaj [1,*]

[1] Department of Chemical and Petroleum Engineering, University of Calgary, 2500 University Dr NW, Calgary, AB T2N 1N4, Canada; milad.kamkar1@ucalgary.ca (M.K.); ssadeghi@ucalgary.ca (S.S.); ehsan.aliabadian@ucalgary.ca (E.A.)

[2] School of Engineering, University of British Columbia, Kelowna, BC V1V 1V7, Canada; mohammad.arjmand@ubc.ca

* Correspondence: u.sundararaj@ucalgary.ca

† Current address: NOVA Chemicals Center for Applied Research, Calgary, AB T2E 7K7, Canada.

Transmission Electron Microscopy

The morphology of synthesized N-CNTs was investigated using TEM images. Figure S1 reveals that the morphology of N-CNTs is a strong function of the synthesis catalyst. N-CNTs grown over Fe and Ni catalysts presented a bamboo-like pattern. The morphological discrepancies of synthesized N-CNTs can be ascribed to different growth mechanisms. The iron particle is recognized to be a decisive player in forming N-CNTs with bamboo-like morphology [[1, 2]]. It has been suggested that nitrogen could also enhance bamboo-like morphology [[3, 4]].

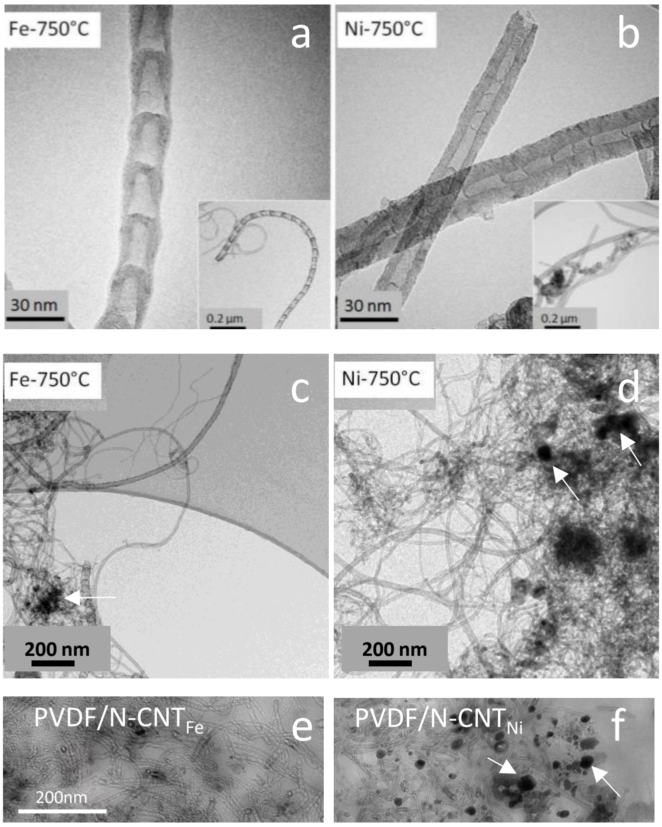

.

Figure S1. : (a)-(d) High-resolution TEM micrographs of N-CNTs grown over Fe and Ni catalysts. (e) and (f) TEM micrographs of nanocomposites containing 2.0wt% nanotubes (arrows show catalyst particles). (a) and (b) Reprinted with permission from Ref. [5]..

Figure S2 presents the average diameter and length of the synthesized N-CNTs. It is evident that N-CNTs grown over Fe catalyst had a much larger diameter than N-based CNT. Measuring the particle size of the growth catalysts displayed that the diameter of N-CNTs is commensurate with the size of catalyst particles. The dissimilarity in the diameter of catalysts can be due to their original size or their distinct affinity to sinter or coalesce at the synthesis temperature. The high reaction temperature and hydrocarbon decomposition (being an exothermic process) cause the formation of supersaturated metal vapor in the reactor, and thus nucleation and metal particle growth by condensation, collision, and sintering [[3, 4]]. This effect is distinct for each type of catalyst and could extremely influence catalyst size distribution and catalyst activity.

The inferior length of $(N\text{-}CNT)_{Ni}$ can be attributed to lower activity of Ni catalyst and/or the existence of a huge amount of nitrogen in its structure. Lower activity of Ni catalysts can be deducted from the inferior carbon purity of its corresponding N-CNT compared to Fe catalysts, as will be displayed by TGA (see Figure S4). On the other hand, the presence of nitrogen can be considered as a key factor to bend, close, and cap N-CNTs, thereby obstructing the growth of N-CNTs. Because of the existence of defects in their crystalline structure, CNTs are believed to break during melt mixing, due to the high applied shear rate [[6]]. Evaluating N-CNTs length distribution in the nanocomposites disclosed that $(N\text{-}CNT)_{Ni}$ and $(N\text{-}CNT)_{Fe}$ experienced 36% and 23% reduction in their original length, respectively. Higher aspect ratio loss in $(N\text{-}CNT)_{Ni}$ can be attributed to its higher nitrogen content, making it more susceptible to breakage.

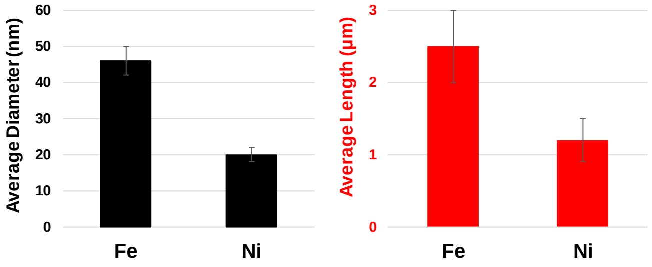

Figure S2. Average diameter and length of N-CNTs synthesized over various catalysts.

X-ray Photoelectron Spectroscopy (XPS).

The quantity of nitrogen content and type of nitrogen bonding can significantly influence morphological, physical, and electronic properties of N-CNTs. Hence, the atomic concentration of nitrogen in synthesized N-CNTs was valued as per the area ratio of XPS N1s and C1s spectra (Table 1). The XPS spectra showed that the atomic percentages of nitrogen within the structure of N-CNTs synthesized over Fe and Ni catalysts were 2.2 and 3.3 at.%, respectively.

There are three common nitrogen bonding configurations in N-CNTs, i.e., quaternary, pyridinic, and pyrrolic. As depicted in Figure S3, the quaternary nitrogen is sp^2 hybridized and directly replaced for a C atom in the hexagonal network. The pyridinic nitrogen is sp^2 hybridized, and has a solitary pair, entering a non-bonding orbital. Pyrrolic nitrogen is sp^3 hybridized, located within a five-membered ring structure, and gives its remaining two electrons to a π orbital, completing the aromatic ring. While the quaternary and pyridinic bondings create side-wall defects, the pyrrolic nitrogen is reported to generate internal cappings, leading to bamboo-like compartments. A fourth type is also reported in the literature (NOX species), known to be an oxidized type of pyridinic nitrogen [[7, 8]].

Table 2 discloses that various types of nitrogen bonding were formed in synthesized N-CNTs, but their ratio is a function of growth catalyst type. It is also seen that N-CNTs grown over Fe catalysts had a relatively lower quantity of pyrrolic nitrogen but a larger amount of NO_X species in comparison

to the (N-CNT)$_{Ni}$. (N-CNT)$_{Ni}$ possessed nearly the same ratios of nitrogen bonding. As already noted, the lower length of N-CNTs grown over Ni catalyst can be related to the larger amount of nitrogen content, particularly pyrrolic nitrogen.

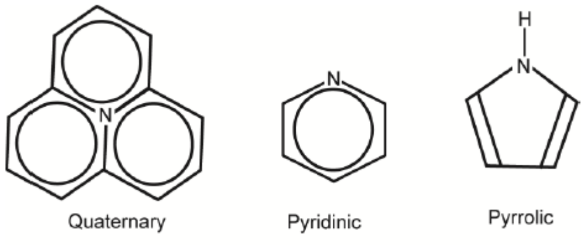

Figure S3. Main types of nitrogen bonding in N-CNTs.

Table S1. Nitrogen content and percentages of various types of nitrogen bonding for N-CNTs synthesized over Fe and Ni catalysts.

Catalyst	Fe	Ni
Nitrogen Content (at.%)	2.2	2.2
Quaternary Nitrogen (%)	46.03	30.22
Pyridinic Nitrogen (%)	14.95	12.32
Pyrrolic Nitrogen (%)	10.58	43.33
NO_X Species (%)	28.43	14.03

Thermogravimetric Analysis (TGA).

Carbon purity and crystallinity of synthesized N-CNTs were investigated employing TGA in the air atmosphere. The first derivative of TGA (DTG) plot illustrates that the inflection points for N-CNTs grown over Fe and Ni catalysts were 605 °C and 574 °C, respectively. It is known that the existence of amorphous carbon and the presence of defects in the morphology of N-CNTs undermine the oxidative stability of these materials [[9]]. The higher inflection point of (N-CNT)$_{Fe}$ signifies its higher crystallinity compared to Ni-based CNT.

In the TGA, the quantity of degraded material signifies carbon purity, and residue after thermal degradation comprises catalyst particles. It was seen that the carbon purities for (N-CNT)$_{Fe}$ and (N-CNT)$_{Ni}$ were 85.1% and 63.9%, respectively. These residues were essentially alumina substrate and metallic particles [[10]]. In this regard, lower carbon purity of (N-CNT)$_{Ni}$ can highly deteriorate the level of conductive network formation, as catalyst particles have much lower surface area than cylindrical CNTs, and even their surface area can drop further owing to probable sintering at the synthesis temperature.

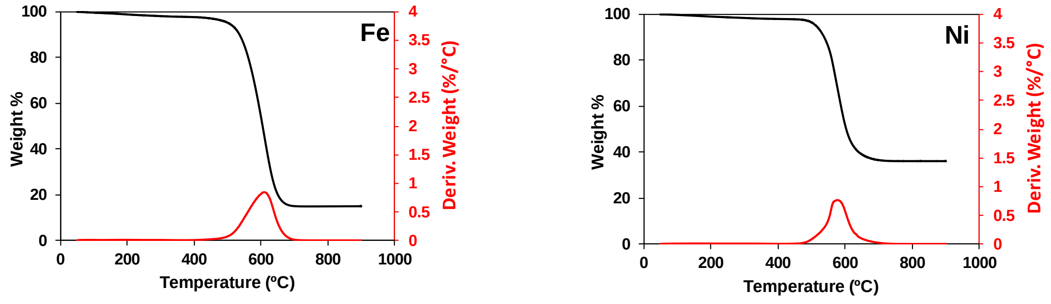

Figure S4. TGA and DTG plots of N-CNTs grown over different catalysts.

Plateau Modulus as a Function of N-CNT Concentration.

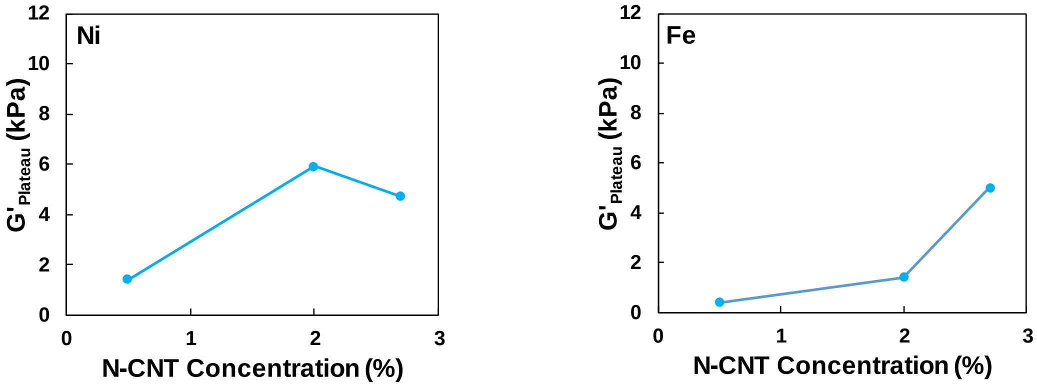

Figure S5. Los-strain plateau modulusfor poorly and well-dispersed CNT/PVDF nanocomposites containing as a function of N-CNT concentration for strain amplitudes of γ_0=0.1 at an angular frequency of ω=0.1rad/s using cone-plate geometry (truncation of 47μm) at 240˚C.

Gap-size Dependence of Stain Amplitude Sweep of Poorly Dispersed System.

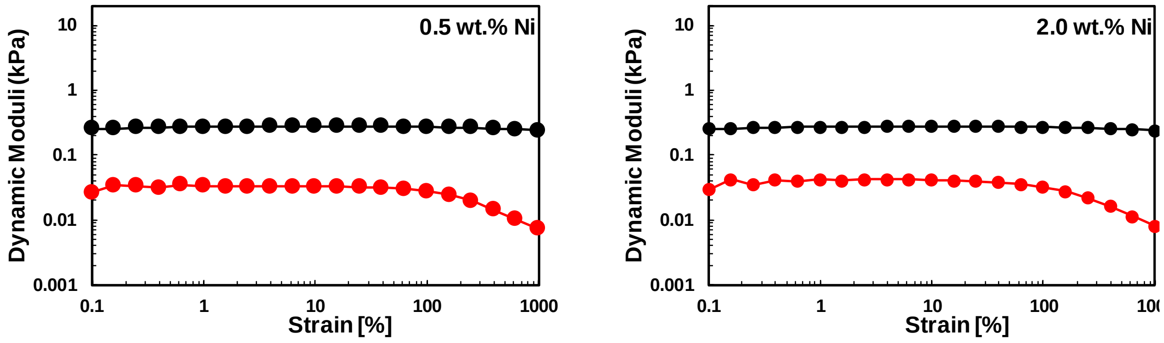

Figure S6. Oscillatory amplitude sweep response of $(N\text{-}CNT)_{Ni}$/PVDF nanocomposites containing 0.5 and 2.0wt.% of carbon nanotubes for strain amplitudes of γ_0=0.1-1000% at an angular frequency of ω=0.1rad/s using a parallel-plate geometry (gap-size of 200μm) at 240˚C.

Schematic of Lissajous-Bowditch Loops.

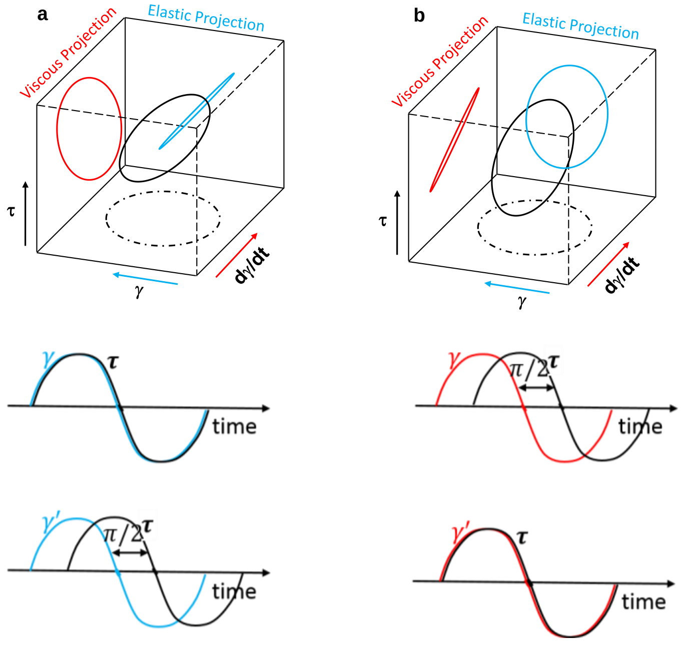

Figure S7. Schematic of Lissajous-Bowditch loops for (a) a perfect elastic and (b) a perfect viscous material on different projections.

Gap-size Dependence of Lissajous Loops.

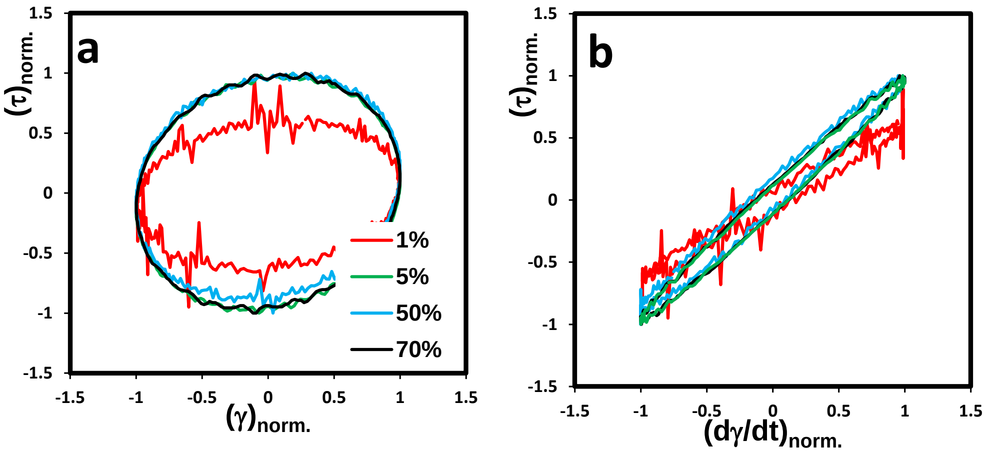

Figure 8. (**a**) Elastic and (**b**) viscous Lissajous-Bowditch loops (N-CNT)Ni/PVDF nanocomposites containing 2.0wt% of carbon nanotubes using a parallel-plate geometry (gap-size of 200µm) at 240 °C. Projections on the elastic (τ-γ) and viscous (τ-$\dot{\gamma}$) planes are presented at strain amplitudes of γ_0 = 1.0, 5.0, 50 and 70% and an angular frequency of ω =0.1rad/s at 240 °C.

Elastic (S) Intra-cycle Nonlinearity Index and Nonlinear Viscoelastic Measures of G'_L and G'_M of Poorly-dispersed (N-CNT)Ni/PVDF Nanocomposite at an Angular Frequency of ω=0.1 and Gap-size of 200µm.

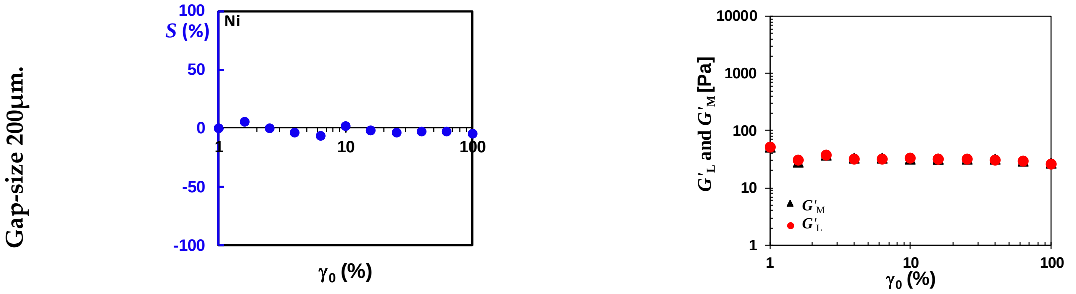

Figure S9. Elastic (S) intra-cycle nonlinearity index and nonlinear viscoelastic measures of G'_L and G'_M as a function of imposed strain amplitude for (N-CNT)Ni/PVDF nanocomposites containing 2.0wt% of carbon nanotubes using a parallel-plate geometry (gap-size of 200μm) at an angular frequency of ω =0.1rad/s at 240 °C.

Elastic (S) Intra-cycle Nonlinearity Index and Nonlinear Viscoelastic Measures of G'_L and G'_M of Poorly-dispersed (N-CNT)Ni/PVDF Nanocomposite at an Angular Frequency of ω=10rad/s.

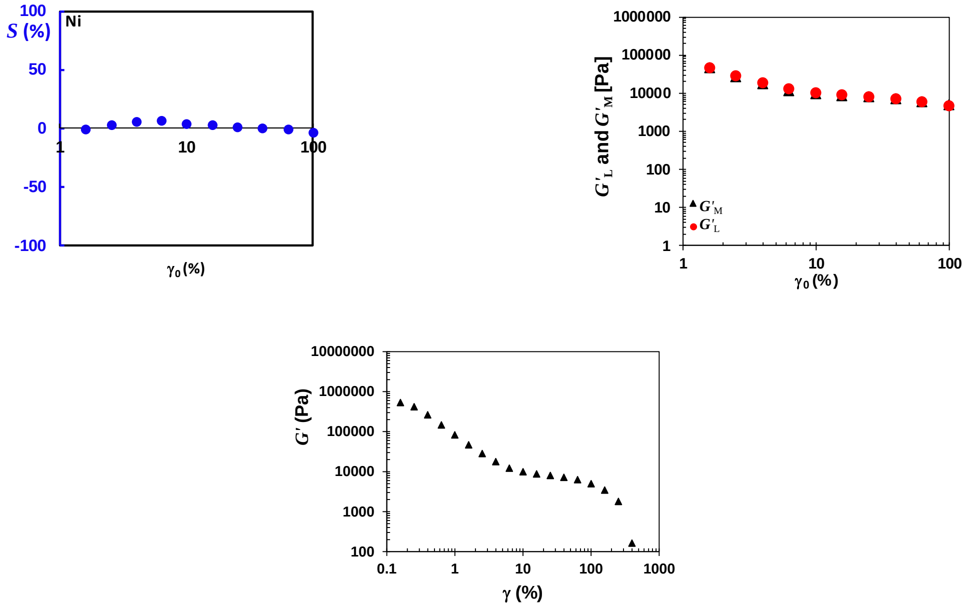

Figure S10. Elastic (S) intra-cycle nonlinearity index, nonlinear viscoelastic measures of G'_L and G'_M and G' as a function of imposed strain amplitude for and (N-CNT)Ni/PVDF nanocomposites using cone-plate geometry (truncation of 47μm) 2.0wt% of carbon nanotubes at an angular frequency of ω =10rad/s at 240 °C.

Chebyshev Coefficients.

To investigate the nonlinear behavior of the samples with a better resolution, quantify the nonlinearity, and find the origin of dissimilarities observed in strain amplitude sweep tests in MAOS region, we extracted Chebyshev coefficients using nonlinear fitting on elastic (σ') and viscous (σ'') stress contributions. This method was introduced by Ewoldt et al. [[11]] in 2008. They suggested a polynomial regression fit to elastic (σ') and viscous (σ'') lines. In their work, they argued the limitation of different polynomial basis functions, such as Jacobi, Laguerre, Hermite, Chebyshev of the first and second kind, and Legendre. Considering the mathematical and physical limitations, e.g., elastic(σ') and viscous (σ'') stresses are orthogonal over a finite domain, they proposed that the set

of Chebyshev polynomials of the first kind is the best choice for fitting the output stress contributions. Then, the authors established a physical interpretation of nonlinear viscoelasticity using Chebyshev coefficients.

Based on this method, a series of Chebyshev polynomials of the first kind in the orthogonal space made up of the input strain and strain-rate can be used to represent the elastic σ' and viscous σ''stress components via the following equations:

$$\sigma'(x\colon \omega, \gamma_0) = \gamma_0 \sum e_n(\omega, \gamma_0)\, T_n(x) \tag{1}$$

$$\sigma''(y\colon \omega, \gamma_0) = \dot{\gamma}_0 \sum v_n(\omega, \gamma_0)\, T_n(y) \tag{2}$$

where $x = \frac{\gamma}{\gamma_0}$ and $y = \frac{\dot{\gamma}}{\dot{\gamma}_0}$ depicts the normalized version of strain and strain-rate, T_n represent the nth-order Chebyshev function and "e" and "v" are Chebyshev weighting coefficients and are a physical interpretation of the elastic and viscous contributions, and have the same units of linear modulus and viscosity, respectively.

The criteria for specification of the physical interpretation of the nonlinearity based on "e" and "v" is defining the concavity of $\sigma'$and σ''. As the magnitude of each Chebyshev coefficient decays monotonically by increasing "n", the third-order Chebyshev coefficients (e_3 and v_3) determine the concavity of the elastic and viscous stress lines. According to these coefficients, the following intra-cycle nonlinear behaviors can be observed: strain-stiffening ($e_3 > 0$), strain-softening ($e_3 < 0$), shear-thickening ($v_3 > 0$), and shear-thinning ($v_3 < 0$).

Figure S11 shows the first order Chebyshev coefficients (e_1 and v_1). Comparing the results of Figure 3 and S11 (compare the arrows with the same color in Figure 3 and S11) reveals that the first order Chebyshev coefficients (e_1 and v_1) in the SAOS region converge to storage modulus (G') and dynamic viscosity ($\frac{G_n''}{\omega} = \eta_n'$), respectively. This conclusion is obtained based on the relationship between the nth-order Chebyshev coefficients and Fourier coefficients, which can be expressed as [[11]]:

$$e_n = G_n' \,(-1)^{(n-1)/2} \tag{3}$$

$$v_n = \frac{G_n''}{\omega} = \eta_n' \tag{4}$$

Hence, in the linear region, the stress response includes just the first harmonic, n=1, and G_1' and G_1'' converge to linear storage and loss moduli. It should be borne in mind that we cannot compare the obtained first-order Chebyshev coefficients with storage modulus G' and dynamic viscosity ($\frac{G_n''}{\omega} = \eta_n'$) at lager strain amplitudes (γ>1%, MAOS region). This is due to the emergence of the higher harmonics in the output stress wave from. Moreover, it is worthwhile mentioning that storage modulus G' and dynamic viscosity ($\frac{G_n''}{\omega} = \eta_n'$) lose their physical meaning in the nonlinear viscoelastic framework.

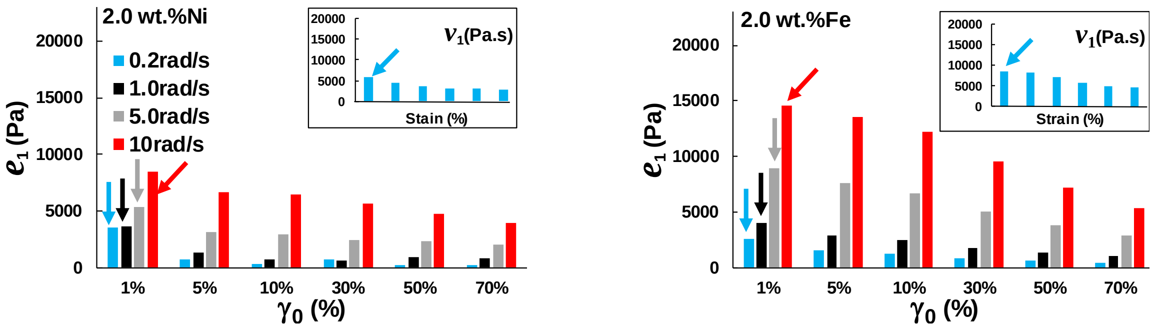

Figure S11. First order elastic Chebyshev coefficient e_1 for N-CNT/PVDF nanocomposites containing 2.0wt% of carbon nanotubes synthesized on different catalysts at strain amplitudes of γ_0= 1.0, 5.0, 10, 30, 50 and 70% and an angular frequency of ω=0.2 (blue), 1.0 (black), 5.0 (grey) and 10 (red) rad/s at 240 °C. The insets represent first order viscous Chebyshev coefficient v_1 for the mentioned strain amplitudes at an angular frequency of ω=0.2rad/s. Data points highlighted by arrows has been compared with data points highlighted with the same color in Figure 3.

Hence, to evaluate the type and extent of nonlinearity and elaborate more on the nonlinear viscoelastic behavior of N-CNT-based nanocomposites in MAOS region, the normalized third-order elastic Chebyshev coefficients (e_3/e_1) are shown in Figure S11. Evaluating the rheological response of nanocomposites containing different types of N-CNT via comparing their e_3/e_1, signifies drastic differences in the origin of their nonlinear viscoelastic behavior. e_3/e_1 are roughly zero for all N-CNT-type nanocomposites in SAOS region (γ<1%, see the linear viscoelastic region in Figure 4, i.e., low-strain plateau moduli). Surpassing the linear region, e_3/e_1 gets positive values and reaches an approximately constant value in the MAOS region for (N-CNT)$_{Fe}$/PVDF. However, (N-CNT)$_{Ni}$/PVDF nanocomposite follows a totally different scenario. This sample experiences the highest elastic nonlinearity (maximum e_3/e_1) at strain amplitude of 5% (after the onset of the nonlinearity at a strain amplitude of 1% where the initial viscoelastic departure from linearity occurs). Then it obeys a decreasing trend, followed by another increasing trend at the strain amplitude of 50%. The elastic nonlinearity in the MAOS region reaches its minimum value at a strain amplitude of 30%. This behavior coincides with the onset of the second plateau in Figure 4, which takes place after the first yielding in the MAOS region. However, we expect an increasing trend in the value of normalized e_3/e_1 after this decay, as the second yielding takes place in Figure 4 by a further increase of input strain amplitude ($\gamma_0 \geq 50\%$). So, e_3/e_1 increased again after a strain amplitude of 30%. The same conclusion can be discerned based on the intra-cycle strain stiffening ratio (see Figure 7).

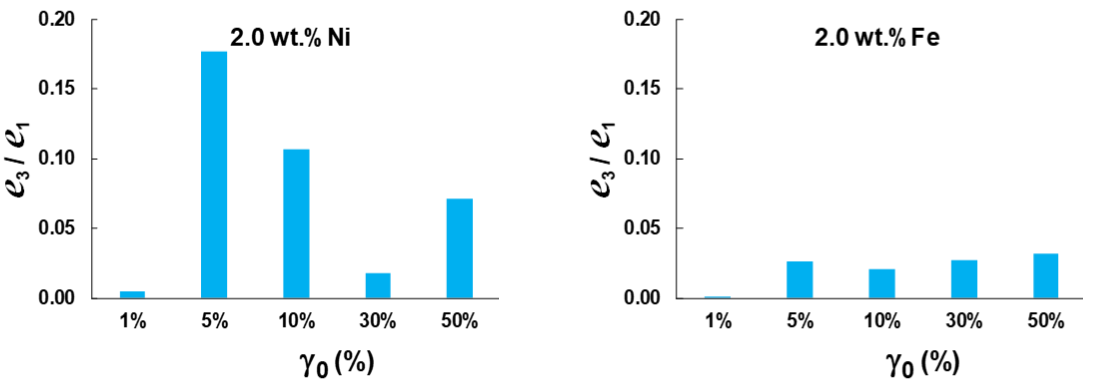

Figure S12. Intra-cycle elastic nonlinearity measures: normalized third-order Chebyshev coefficient e_3/e_1 for CNT/PVDF nanocomposites containing 2.0wt% of carbon nanotubes synthesized on different catalysts at strain amplitudes of γ_0= 1.0, 5.0, 10, 30, and 50% and an angular frequency of ω=0.1rad/s at 240 °C.

References.

1. Choi, H.C., J. Park, and B. Kim, *Distribution and structure of N atoms in multiwalled carbon nanotubes using variable-energy X-ray photoelectron spectroscopy.* The Journal of Physical Chemistry B, 2005. **109**(10): p. 4333-4340.

2. Lee, Y.T., et al., *Temperature-dependent growth of carbon nanotubes by pyrolysis of ferrocene and acetylene in the range between 700 and 1000 C.* Chemical physics letters, 2003. **372**(5-6): p. 853-859.
3. Lin, C.H., et al., *The role of nitrogen in carbon nanotube formation.* Diamond and related materials, 2003. **12**(10-11): p. 1851-1857.
4. Chang, H.L., C.H. Lin, and C.T. Kuo, *Field emission, structure, cathodoluminescence and formation studies of carbon and Si–C–N nanotubes.* Diamond and related materials, 2002. **11**(3-6): p. 793-798.
5. Ameli, A., et al., *Effects of synthesis catalyst and temperature on broadband dielectric properties of nitrogen-doped carbon nanotube/polyvinylidene fluoride nanocomposites.* Carbon, 2016. **106**: p. 260-278.
6. Arjmand, M., et al., *An innovative method to reduce the energy loss of conductive filler/polymer composites for charge storage applications.* Composites Science and Technology, 2013. **78**: p. 24-29.
7. Ghosh, K., et al., *Controllable growth of highly N-doped carbon nanotubes from imidazole: a structural, spectroscopic and field emission study.* Journal of Materials Chemistry, 2010. **20**(20): p. 4128-4134.
8. Maldonado, S., S. Morin, and K.J. Stevenson, *Structure, composition, and chemical reactivity of carbon nanotubes by selective nitrogen doping.* Carbon, 2006. **44**(8): p. 1429-1437.
9. Li, H., et al., *Thermogravimetric analysis and TEM characterization of the oxidation and defect sites of carbon nanotubes synthesized by CVD of methane.* Materials Science and Engineering: A, 2008. **473**(1-2): p. 355-359.
10. Mirkhani, S.A., et al., *Impact of synthesis temperature on morphology, rheology and electromagnetic interference shielding of CVD-grown carbon nanotube/polyvinylidene fluoride nanocomposites.* Synthetic Metals, 2017. **230**: p. 39-50.
11. Ewoldt, R.H., A. Hosoi, and G.H. McKinley, *New measures for characterizing nonlinear viscoelasticity in large amplitude oscillatory shear.* Journal of Rheology, 2008. **52**(6): p. 1427-1458.
